# The difference in the effect of methadone and protracted abstinence on the coupling among key large-scale brain networks of individuals with heroin use disorder: A resting-state fMRI study

**DOI:** 10.1017/S0033291725101451

**Published:** 2025-08-26

**Authors:** Jiajie Chen, Ning Wu, Long Jin, Jia Zhu, Yongbin Li, Zhidong Wang, Fan Wang, Wei Wang, Wei Li, Qiang Li

**Affiliations:** 1 Department of Radiology, Tangdu Hospital, Fourth Military Medical University, Xi’an, China; 2 Department of Radiology, The Eighth Medical Center of the People’s Liberation Army General Hospital, Beijing, China; 3 Department of Nuclear Medicine, Tangdu Hospital, Fourth Military Medical University, Xi’an, China; 4 Department of Radiology, Xi’an No. 1 Hospital, Xi’an, China

**Keywords:** default mode network, heroin, magnetic resonance imaging, methadone, protracted abstinence, resource allocation index, salience network

## Abstract

**Background:**

Methadone maintenance treatment (MMT) and protracted abstinence (PA) effectively reduce the craving for heroin among individuals with heroin use disorder (HUD). However, the difference in their effects on brain function, especially the coupling among the large-scale brain networks (default mode [DMN], salience [SN], and executive control [ECN] networks), remains unclear. This study analyzed the effects of the MMT and PA on these networks and the predictive value of the bilateral resource allocation index (RAI) for craving for heroin.

**Methods:**

Twenty-five individuals undergoing the MMT, 22 undergoing the PA, and 51 healthy controls underwent resting-state functional magnetic resonance imaging (rs-fMRI). Independent component analysis identified the ECN, DMN, and SN. The SN-ECN and SN-DMN connectivity and the bilateral RAI were evaluated. The relationships between network coupling and clinical and psychological characteristics were analyzed. The multiple linear regression model identified significant variables for predicting craving scores.

**Results:**

The MMT group showed significantly stronger SN-left ECN (lECN) coupling and left RAI than the PA group. In the MMT group, SN-lECN connectivity and bilateral RAI were positively correlated with the total methadone dose. In both treatment groups, SN-right ECN (rECN) connectivity and right RAI were negatively correlated with craving. The models revealed that the bilateral RAI and the MMT and PA were associated with the craving.

**Conclusions:**

The MMT enhances SN-lECN coupling and the left RAI more than the PA, possibly due to higher control modulation. The RAI could help predict heroin craving in individuals with HUD undergoing either treatment program.

## Introduction

Heroin use disorder (HUD) is a complex psychiatric condition characterized by drug-craving, tolerance, withdrawal symptoms, compulsive drug-taking, and relapse (Browne et al., 2023). The 2024 World Drug Report reveals that in 2022, opioid drug usage reached 60 million individuals, accounting for approximately 1.2% of the global population (https://www.unodc.org/unodc/). Heroin users in Europe use it as a primary or secondary drug (Roman-Urrestarazu et al., 2018). The situation is more serious in North America (Milano et al., 2017). Drug addiction has led to a surge in criminal activities and healthcare expenditures, causing significant concerns domestically in China and globally. Research indicated that effectively reducing cravings for heroin and preventing relapse are central issues when treating heroin dependence (Chen et al., 2022; Wang et al., 2021).

Methadone treatment is the gold standard for heroin withdrawal treatment. Methadone maintenance treatment (MMT) and protracted abstinence (PA) are important therapeutic strategies for addiction in China, aiming to markedly reduce or eliminate the use of illicit opioids and restore social roles (McLellan, McKay, Forman, Cacciola, & Kemp, 2005). MMT remains the standard against which other medications, including buprenorphine and naltrexone, are compared (Bell & Strang, 2020). However, dropout from MMT programs is common (Zhang et al., 2015), and inadequate methadone doses compromise treatment effectiveness (Faggiano, Vigna-Taglianti, Versino, & Lemma, 2003). The PA program encompasses detoxification, comprehensive physical healthcare, and psychological and behavioral therapy (Chen et al., 2019). The high relapse rate following the PA program remains perplexing (Su et al., 2015). Although the MMT and PA programs effectively diminish the craving for heroin among individuals with HUD, it remains uncertain which program is the optimal treatment strategy for promoting heroin abstinence and how each impacts the brain’s functional activity.

To date, a few studies have demonstrated the different effects the PA and MMT programs have on brain functions in individuals with HUD. Wei et al. (2020) found that the brain responses of individuals under MMT were significantly greater than those under PA during a drug cue-reactivity task, particularly in regions such as the caudate, hippocampus, posterior cingulate cortex, and inferior parietal lobule. Individuals undergoing long-term MMT (approximately 12 months) exhibited persistently higher subjective craving and brain activation during exposure to heroin-related cue tasks than those receiving PA, including the caudate, hippocampus, anterior cingulate cortex, middle cingulate cortex, inferior parietal lobule, amygdala, etc. (Wei, Li, et al., 2021). PA is more effective in reducing the salience value of heroin cues in individuals with HUD during cue-reactivity tasks (Wei, Chen, et al., 2021). Despite recent advances, our understanding of the differences between the MMT and PA programs in HUD-associated brain function characteristics remains limited.

Resting-state functional magnetic resonance imaging (rs-fMRI) is a robust neuroimaging tool to investigate spontaneous functional activity among spatially distributed brain regions without any controlled experimental paradigm (Khosla, Jamison, Ngo, Kuceyeski, & Sabuncu, 2019). It can potentially analyze the brain’s functional organization in individuals with HUDs and how the MMT and PA programs influence it. Evidence from previous neuroimaging studies has demonstrated the crucial association between addiction and key neurobiological mechanisms, particularly large-scale functional networks such as the executive control (ECN), default mode (DMN), and salience (SN) networks (Jin et al., 2022; 2023). The DMN mainly consists of the ventromedial prefrontal cortex (vmPFC), posterior cingulate cortex (PCC), precuneus, and bilateral angular gyrus (AG) (Garner & Keller, 2022). The DMN is frequently activated in response to self-referential mental processes, such as autobiographical memory and prospective thinking (Gusnard, Akbudak, Shulman, & Raichle, 2001) and deactivated during tasks requiring cognitive efforts. The ECN and DMN activities are negatively correlated. The highest ECN activation is during decision-making tasks and problem-solving activities (Uddin et al., 2023). This network has crucial nodes with the dorsolateral PFC (dlPFC) and posterior parietal cortex. The SN comprises the bilateral anterior insula, the anterior cingulate cortex, and the medial PFC (mPFC) (Cai, Chen, Szegletes, Supekar, & Menon, 2015). Its function is a dynamic switch between self-focused attention and introspection facilitated by the DMN and task-related or -directed attention toward external stimuli maintained by the ECN (Seeley, 2019). A large-scale functional network model emphasizes the role of the SN in directing attentional resources toward the DMN and ECN, an essential process for regulating complex cognitive and behavioral control (Cai et al., 2016). The triple network model of psychopathology suggests that abnormal functional organization and dynamic cross-network connectivity among these networks might underlie various psychiatric symptoms (Schimmelpfennig, Topczewski, Zajkowski, & Jankowiak-Siuda, 2023).

The resource allocation index (RAI) was proposed as a measure to evaluate the SN-ECN and SN-DMN connectivity strength as a measure of brain function. A reinforced SN activity could increase intrinsic functional connectivity between the DMN and ECN, efficiently allocating attentional resources toward task-relevant stimuli while suppressing attention toward distraction and self-related mental processes (Seeley et al., 2007). Conversely, a low RAI score might indicate a tendency for inward-directed attention, suggesting attentional deficits (Choi, Jeong, Lee, & Go, 2013). Altered coupling among large-scale networks has been proposed as a potential neural mechanism for HUD (Li et al., 2018; Zhang et al., 2024). However, no studies have explored the differential effects the MMT and PA programs have on coupling among large-scale brain networks.

Our previous research demonstrated that individuals undergoing the MMT program exhibited elevated SN-bilateral ECN coupling (Chen et al., 2022). The PA program was shown to improve the SN-bilateral ECN, internal of left ECN (lECN), and right ECN (rECN)-DMN connectivity in individuals with HUD (Chen et al., 2021). We computed the RAI to assess the integrity of interactions among the three key large-scale networks. The primary goal of this study was to analyze disparities between MMT and PA in coupling among the three key large-scale brain networks and RAI in participants with HUD under the resting-state condition. Furthermore, we investigated the associations between post-intervention heroin-related craving, the treatment type, and the bilateral RAI scores.

## Methods

### Participants

Patients undergoing MMT were recruited from a heroin treatment program in Baqiao District, Xi’an, China, from January 2013 to December 2016. Patients undergoing PA were recruited at the Lantian Rehabilitation Center in Xi’an, China, during the same period. The inclusion criteria for both groups were as follows: (1) diagnosed with opioid use disorder according to the Diagnostic and Statistical Manual of Mental Disorders (Fifth Edition); (2) was on a stable methadone dosage for at least 3 months in a methadone clinic or underwent a PA program for at least 3 months; and (3) was of the Han ethnicity, right-handed based on a handedness questionnaire, and aged 20–50 years. Healthy volunteers in the community around the Tangdu Hospital were recruited for the healthy control (HC) group. Participants were excluded if they met any of the following criteria: (1) had substance dependence disorders other than tobacco and heroin; (2) had a history of brain disorders such as mental illness or brain injury; and (3) had contraindications for magnetic resonance imaging (MRI) scanning. The study enrolled 25 patients undergoing the MMT program, 22 undergoing the PA program, and 60 HCs. The study protocol was approved by the ethical committee of the Tangdu Hospital (no. TDLL-2016123). Informed consent was obtained from all participants, who voluntarily consented and who were free to withdraw from the study at any time without affecting their usual medical treatment.

### Craving assessment

Heroin-related craving, defined as the intensity of the desire to use heroin, was evaluated using an event-related task paradigm with images. The images consisted of heroin-related (heroin, syringes, etc.) and neutral (roads, sky, buses, etc.) stimuli. These images were presented in a pseudorandom order through a computer for approximately 6 minutes. Subsequently, the participants’ subjective psychological craving for heroin was assessed using a self-rating visual analog scale. The scale ranged from 0 (no subjective craving for heroin) to 10 (extremely strong and very difficult to control subjective craving for heroin). The craving was assessed after the MRI scanning.

### MRI acquisition

A negative urine test for morphine, methamphetamine, ketamine, cocaine, and ecstasy was required on the day of the MRI scan. Individuals undergoing the MMT program took methadone after the MRI scan. The MRI scans were performed using a 3T scanner (Signa Excite HDx, General Electric) with a standard eight-channel phase-array head coil at the Tangdu Hospital. During the MRI scanning procedure, the participants were instructed to remain awake and fixate their gaze on a white cross on a black screen. A simulated rs-fMRI scan was performed for approximately 1 minute without data acquisition to facilitate adaptation to the MRI scanning environment. Subsequently, rs-fMRI data were collected during the 5-minute formal scan using a gradient echo-planar imaging sequence (repetition time, 2,000 ms; echo time, 30 ms; field of view, 256 × 256 mm; image matrix, 64 × 64; slice thickness, 4 mm with no gap between slices; a total of 150 volumes). High-resolution T1-weighted imaging data were acquired using a three-dimensional fast spoiled gradient sequence (repetition time, 7.8 ms; echo time, 3.0 ms; inversion time, 450 ms; flip angle, 20°; field of view, 256 × 256 mm; matrix, 256 × 256; slice thickness, 1 mm with no gap between slices; a total of 166 slices). Two experienced neuroradiologists assessed all MRI scans to exclude any brain abnormalities.

### Image data processing

#### Preprocessing

The functional and structural images were preprocessed with DPABI (http://rfmri.org/dpabi) and SPM12 (www.fil.ion.ucl.ac.uk/spm) software, implemented in MATLAB 8.1 (www.mathworks.com). Preprocessing steps of the rsfMRI data included slice-timing correction, motion realignment, co-registration of the anatomical and functional images, segmentation, nuisance covariate regression (including Friston 24 head motion parameters and cerebrospinal fluid and white matter signal noises), normalization, Gaussian smoothing with a kernel size of 6 mm at full width and half maximum, detrending, and bandpass filtering between 0.01 and 0.1 Hz. No participants were excluded due to head movements as these did not exceed 2.5 mm or 2.5°.

#### Identification of the three large-scale networks

**Figure 1.**
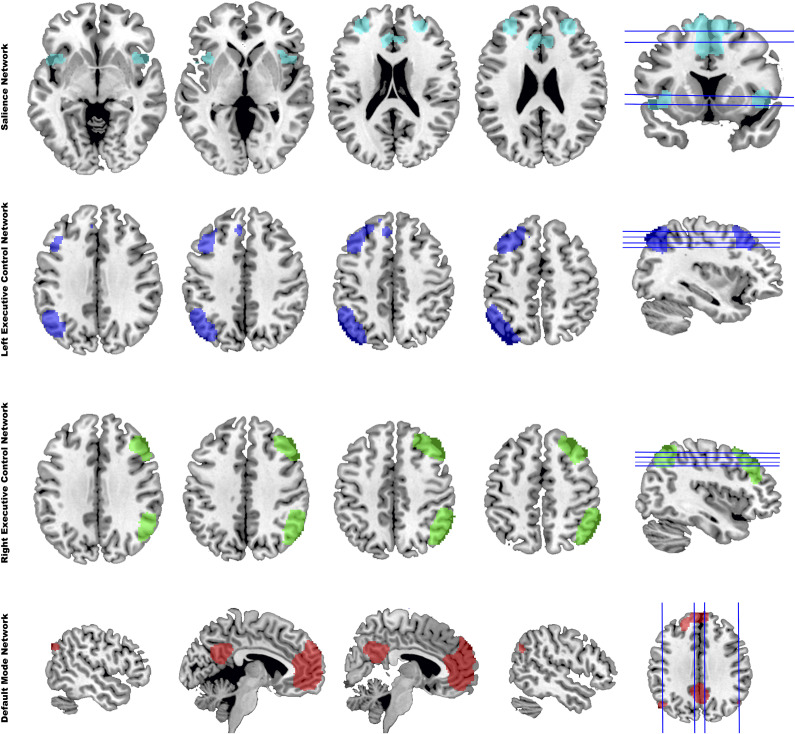
The four composite maps were generated by group-independent component analysis of resting-state fMRI data, identified as salience network (SN), left executive control network (lECN), right executive control network (rECN), and default mode network (DMN).

We selected regions of interest from the DMN, SN, and bilateral ECN. The definitions of these networks were based on previous studies (Seeley et al., 2007; Shirer, Ryali, Rykhlevskaia, Menon, & Greicius, 2012; Williams, 2016). The DMN comprises the vmPFC, PCC, and bilateral AG (Fox et al., 2005). The ECN includes the dlPFC and posterior parietal cortex (Niendam et al., 2012). The SN comprises the bilateral anterior insula, dorsal anterior cingulate cortex, and mPFC (Menon, 2011) (Figure 1).

#### Network-to-network connectivity analysis

The functional connectivity (FC) among three core large-scale networks was calculated by first extracting an average time series for each network (SN, rECN, lECN, and DMN) as a reference for each participant. Subsequently, Pearson’s correlation coefficient estimated the FC based on the average time series of each network in each participant. To improve the normal distribution for statistical comparisons, individual correlation coefficients were transformed using Fisher’s *Z* transformation. The analysis of variance (ANOVA) detected inter-group differences in FC among the SN, bilateral ECN, and DMN. The false discovery rate (FDR) correction was employed when multi-statistical tests were simultaneously performed on all FCs. The threshold for multiple comparisons correction was set as *P* = 0.05.

#### Resource allocation index analysis

The RAI was computed for each participant to quantify the connectivity strength in the SN-ECN and SN-DMN pairs, as previously described (Reese et al., 2019). The RAI in the resting state was evaluated using the formula: RAI = *f* (CC_SN_, _ECN_) *– f* (CC_SN, DMN_), where CC_SN_, _ECN_ and *CC*_SN, DMN_ represent Pearson’s correlation coefficient between the time series of the two network pairs. The function *f* (*x*) denotes Fisher’s *Z*-transformation of correlation coefficients (CCs). Higher RAI values suggest a stronger coupling between SN and the bilateral ECN and a weaker coupling between SN and DMN. High RAI values imply that the action of SN tends to prioritize external stimuli processing over internal attention. The threshold for multiple comparisons correction was set at a significant level of *P* = 0.05. If ANOVA detected significant inter-group differences in RAI, the FDR correction was used at *P* <0.05.

### Relationship between the network model and clinical characteristics

To investigate the relationship between clinical characteristics and the network model, we computed Pearson’s correlation coefficients to assess the association between the FC strength or bilateral RAI and heroin-related craving scores or methadone doses in the MMT and/or PA groups (FDR corrected at *P* < 0.05).

Multiple linear regression analysis was conducted to examine the association between post-intervention heroin-related craving scores and the observed change, including treatment type (PA or MMT) and bilateral RAI. The regression equation is shown in the following equation:

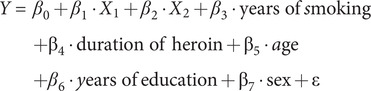

where *Y* is the post-intervention heroin-related craving score, 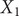 is the treatment type (the MMT or PA program), 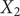 is the RAI value, and ε is the random variation in the model.

## Results

### Participant characteristics

The three groups were similar in sex, age, educational level, smoking, and head motion (*P* > 0.05). The MMT and PA groups had similar baseline characteristics, including heroin use, duration of treatment, and level of craving (*P* > 0.05). The demographic and clinical characteristics of the participants are summarized in Table 1.

**Table 1.** Demographic and clinical characteristics of the three groups

Participant characteristics	HC (*n* = 51)	MMT (*n* = 25)	PA (*n* = 22)	x^2^/F/t	*P*
Sex (male/female)^a^	49/2	25/0	20/2	2.477	0.290
Age (years)^b^	35.1 ± 7.7	35.2 ± 7.7	35.9 ± 6.2	0.095	0.910
Education (years)^b^	10.2 ± 2.3	10.72 ± 2.2	9.7 ± 2.8	0.833	0.438
Cigarette smoking (years)^b^	15.2 ± 6.8	16.8 ± 4.6	15.7 ± 5.9	0.770	0.466
Cigarettes per day (*n*)^b^	17.1 ± 9.2	18.1 ± 5.2	19.1 ± 7.4	0.537	0.586
MeanFD_Power^b^	0.1 ± 0.1	0.1 ± 0.01	0.1 ± 0.01	1.523	0.223
MeanFD_Jenkinson^b^	0.1 ± 0.04	0.1 ± 0.03	0.07 ± 0.04	1.628	0.202
Heroin use (months)^b^	-	85.3 ± 65.8	94.3 ± 58.1	–0.497	0.621
Heroin used per day (g)^c^	-	0.5 ± 0.4	0.7 ± 0.5	–1.589	0.119
Methadone used per day (mL)	-	37.2 ± 17.2	-	-	-
Treatment duration (months)^c^	-	5.7 ± 1.1	6.3 ± 1.1	–1.348	0.184
Heroin-related craving score^c^		2.7 ± 1.1	2.2 ± 1.2	1.266	0.214

*Note*: Values are presented as means ± standard deviation.

a Chi-squared test.

b Analysis of variance.

c Two samples *t*-test.

### Effects of treatment on inter-network connectivity

**Figure 2.**
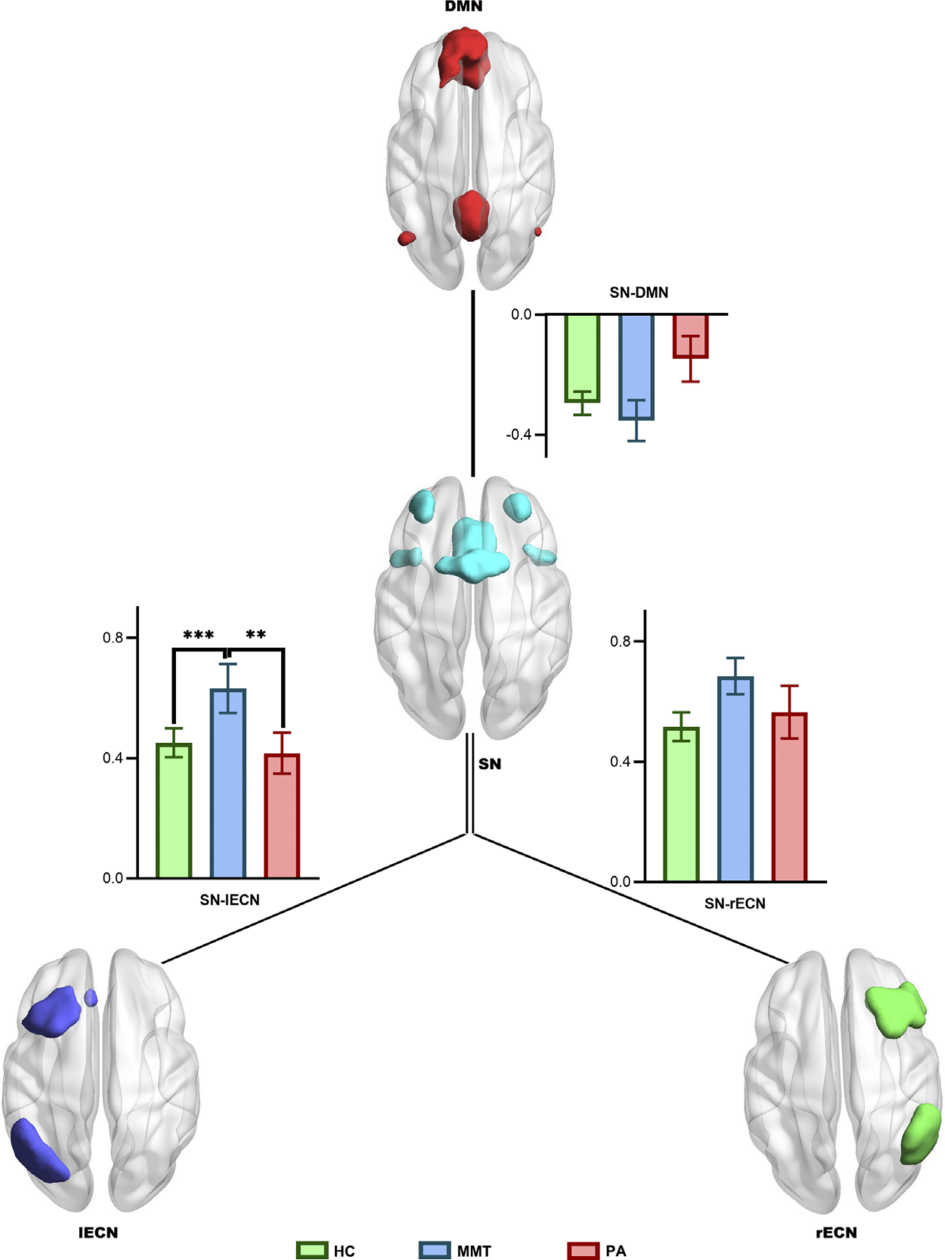
The spatial maps and connectivity graphs compare the interrelationships in the three networks among the healthy control (HC), methadone maintenance treatment (MMT), and protracted abstinence (PA) groups. ** *P* < 0.01; *** *P* < 0.001.

We established a model to compare the effects of the treatment program on brain inter-network connectivity in individuals with HUD and the same treatment duration. This analysis revealed significant differences in connectivity between SN and lECN among the HC, MMT, and PA groups (*F* = 4.455, *P* = 0.014, FDR corrected). Analysis revealed that the MMT group had significantly stronger SN-lECN connectivity than the HC and PA groups. However, the three groups had similar SN-DMN and SN-rECN connectivity (*F* = 0.177, *P* = 0.838 and *F* = 1.683, *P* = 0.191, respectively; Table 2; Figure 2).

**Table 2.** The network-coupling characteristics of the three groups

	HC (*n* = 51)	MMT (*n* = 25)	PA (*n* = 22)	*F*	*P*
SN-lECN	0.376 ± 0.041	0.546 ± 0.049	0.324 ± 0.056	4.455	0.014
SN-rECN	0.516 ± 0.047	0.525 ± 0.065	0.569 ± 0.084	0.177	0.838
SN-DMN	–0.295 ± 0.038	–0.353 ± 0.068	–0.287 ± 0.031	1.683	0.191
Right RAI	0.813 ± 0.079	0.862 ± 0.101	0.76 ± 0.136	0.188	0.829
Left RAI	0.672 ± 0.07	0.899 ± 0.101	0.76 ± 0.105	3.534	0.033

*Note:* Values are presented as means ± standard deviation.

Abbreviations: lECN, left executive control network; RAI, resource allocation index; rECN, right executive control network; SN, salience network.

### Effects of treatment on the bilateral RAI scores

**Figure 3.**
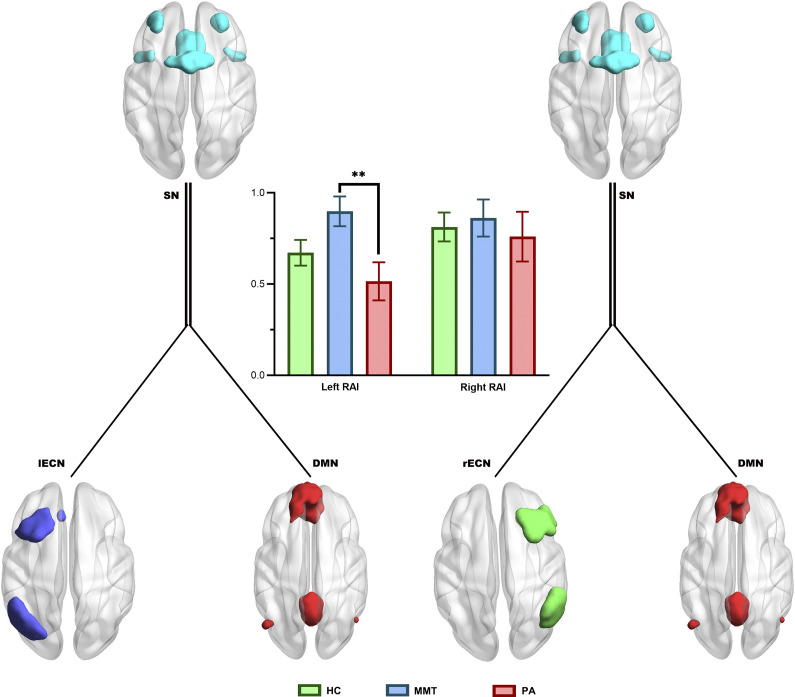
Group differences in the bilateral resource allocation index (RAI) scores. HC, healthy control; MMT, methadone maintenance treatment; PA, protracted abstinence. **, *P* < 0.01.

We analyzed the mediating role of SN as an RAI between the external (bilateral ECN) and internal (DMN) modes in the HC, MMT, and PA groups. Our results revealed no significant differences in the right RAI scores among the three groups (*F* = 0.188, *P* = 0.829). However, a significant difference was observed in the left RAI scores among the three groups (*F* = 3.534, *P* = 0.033, FDR corrected). The post hoc analysis revealed that the MMT group had a significantly higher left RAI score than the PA group (Table 2; Figure 3).

### Linear regression analysis

Linear regression models revealed that the right RAI score (β = –1.253; *t* = –4.106; *P* < 0.001; 95% confidence interval [CI], –1.875 to –0.632; variance inflation factor = 1.051) and treatment type (β = –0.709; *t* = –2.286; *P* < 0.029; 95% CI, –1.342 to –0.077; variance inflation factor = 1.051) were negatively associated with post-intervention heroin-related craving, explaining 37.1% of the variance (*F* = 9.431; *P* = 0.001; *R* ^2^ = 0.371; Durbin Watson = 1.982). Furthermore, the left RAI (β = –1.093; *t* = –3.325; *P* < 0.002; 95% CI, –1.763 to –0.424; variance inflation factor = 1.264) and treatment type (β = –0.979; *t* = –2.700; *P* < 0.011; 95% CI, –1.717 to –0.240; variance inflation factor = 1.264) were negatively associated with post-intervention heroin-related craving, explaining 28% of the variance (*F* = 6.410; *P* = 0.005; *R* ^2^ = 0.286; Durbin Watson = 2.027).

### Relationship between network connections and clinical variables

**Figure 4.**
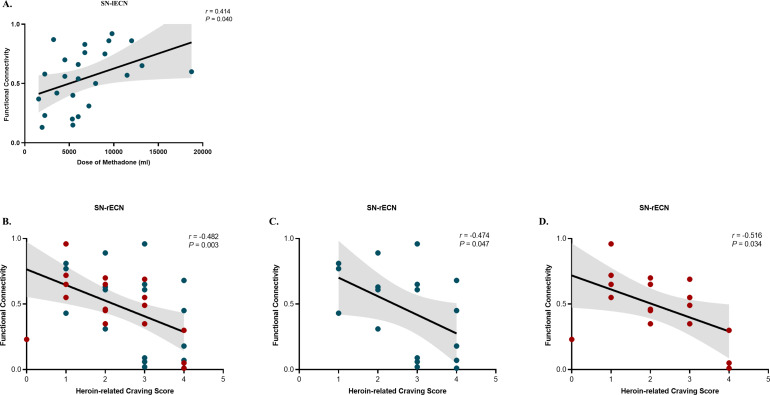
Correlation between SN-lECN connectivity and total methadone dose in the MMT group (A). Correlation between SN-rECN connectivity and post-intervention heroin-related craving scores in the MMT and PA groups together (B), as well as within each group alone (C, MMT group; D, PA group). SN, salience network; lECN, left executive control network; rECN, right executive control network; MMT, methadone maintenance treatment; PA, protracted abstinence. Teal dots denote individuals undergoing MMT program. Red dots denote individuals undergoing PA program.

Pearson’s correlation analysis showed a positive association between the FC of the SN-lECN and the methadone dose (*r* = 0.414; *P* = 0.040; Figure 4A) in the MMT group. An association between the connectivity of the SN-rECN and post-intervention heroin-related craving was observed in the MMT and PA groups together (*r* = –0.482; *P* = 0.003; *n* = 35; Figure 4B), as well as within each group alone (MMT: *r* = –0.474; *P* = 0.047; *n* = 18; Figure 4C; PA: *r* = –0.516; *P* = 0.034; *n* = 17; Figure 4D). The significance threshold for multiple comparisons after FDR correction was *P* = 0.047.

**Figure 5.**
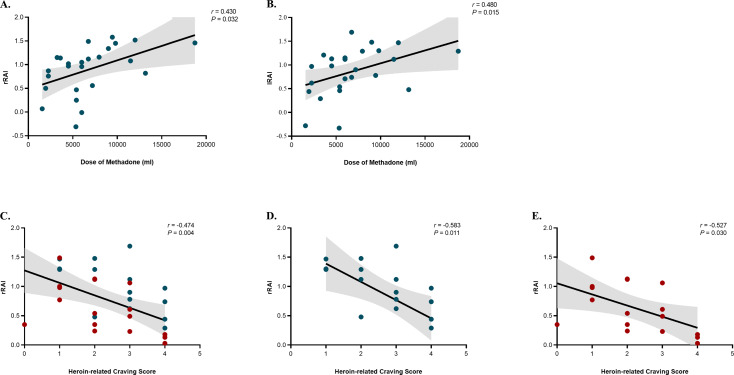
Correlation between the bilateral RAI and the total methadone dose (A, right; B, left) in the MMT group. Correlation between the right RAI and post-intervention heroin-related craving scores in the MMT and PA groups together (C), as well as within each group alone (D, MMT group; E, PA group). RAI, resource allocation index; MMT, methadone maintenance treatment; PA, protracted abstinence. Teal dots denote individuals undergoing MMT program. Red dots denote individuals undergoing PA program.

We also found a positive correlation between the bilateral RAI scores and the methadone dose in the MMT group (right: *r* = 0.430; *P* = 0.032; Figure 5A; left: *r* = 0.480; *P* = 0.015; Figure 5B). Furthermore, high right RAI scores were associated with a decreased heroin-related craving in the PA and MMT groups together (*r* = –0.474; *P* = 0.004; *n* = 35; Figure 5C), as well as within each group alone (MMT: *r* = –0.583, *P* = 0.011, *n* = 18; Figure 5D; PA: *r* = –0.527, *P* = 0.030, *n* = 17; Figure 5E). The significance threshold for multiple comparisons after FDR correction was *P* = 0.032.

## Discussion

This study evaluated differences in the effect of MMT and PA on the SN-ECN and SN-DMN inter-network relations using the RAI scores. The left RAI scores in the MMT group were significantly higher than in the PA group. Further analysis revealed a significantly stronger SN-lECN connectivity in the MMT group than that in the PA group. These were negatively correlated with post-intervention heroin-related cravings in both groups. The study further revealed that the MMT program and higher RAI scores had a stronger predictive power for craving for heroin among individuals with HUD than the PA program. Our findings also indicated that treatment type (MMT or PA) and the RAI score were important predictors of craving for heroin.

### MMT exerts more modulating control over network coupling than PA

Our results showed that the left RAI score in individuals with HUD was significantly higher in the MMT program than in the PA program. Analysis of inter-network connectivity revealed that SN-lECN in the PA group was notably weaker than in the MMT group. Our previous findings suggested that long-term MMT for individuals with HUD could increase their FC between the SN and bilateral ECN (Chen et al., 2022). Long-term PA improved the coupling between SN and ECN in individuals with heroin addiction and elicited drug-cue-induced craving (Chen et al., 2021). A quantitative RAI that integrates inter-network connectivity between SN and bilateral ECN/DMN provides comprehensive information about self-awareness and cognitive control (Lerman et al., 2014). We found that the MMT group had higher RAI scores and SN-ECN connectivity than the PA group, implying that MMT could enhance individuals’ ability to regulate their response to external stimuli and improve self-control behavior. The heightened SN-lance connectivity in the MMT group could also imply a higher SN to allocate additional resources toward regulating top-down cognitive control that inhibits or prevents craving (Zhang et al., 2017). Our study suggested that MMT was useful in exerting stronger control than PA in patients undergoing treatment for HUD, particularly in modulating large-scale brain network coupling.

### MMT and PA protocols and the RAI scores could predict craving

Our multiple linear regression model demonstrated a significant association between higher bilateral RAI scores in the MMT group and lower craving scores among individuals with HUD. Tabatabaei-Jafari et al. (2014) found that although MMT and PA successfully helped individuals with HUD to reduce heroin craving, the underlying neural mechanism of treatment essentially differed in the brain network. A review showed that the effectiveness of therapeutic interventions (methadone, naltrexone, etc.) was associated with the improvement of executive control networks in individuals with HUD (Cabrera et al., 2016). A lower RAI score could predict abstinence-induced craving for smoking (Lerman et al., 2014). The crucial aspect of the RAI model lies in allocating salience activity to external stimuli and internal mental events (Menon & Uddin, 2010). The coupling among large-scale networks could help explain the mechanisms of addiction and, more importantly, serve as focal points for developing biomarkers to predict diagnostic and treatment outcomes (Fedota & Stein, 2015). Our findings further expanded the understanding of therapeutic approaches to HUD, suggesting that treatment strategies and the novel RAI could predict the intensity of heroin-related craving following intervention.

### The coupling among key large-scale networks is linked to craving

We found that SN-leech connectivity and the bilateral RAI scores positively correlated with the methadone dose in the MMT group. Interestingly, we also observed a negative correlation between SN-recent connectivity and the right RAI scores and post-intervention heroin-related craving in the MMT and PA groups together and in each alone. These findings supported neurobiological models of HUD, highlighting the importance of SN-ECN connectivity in evaluating treatment effectiveness in individuals with HUD. Our findings are supported by a previous study that provided evidence for the interaction among cue reactivity, craving, and cognitive control in substance use disorders (Goldstein & Volkow, 2011). Zhang et al. (2017) found that the right RAI score was negatively associated with craving in Internet gaming disorder. Functional connectivity between the dlPFC (a key region of ECN) and the insula (a key region of SN) was important for inhibiting psychological cravings in individuals with HUD undergoing the PA program (Zhang et al., 2022). Liao et al. (2014) suggested that MMT facilitates inhibitory control function and mitigates risky behaviors in HUDs. A similar study suggested that individuals with HUDs receiving MMT showed higher functional decision-making under objective risk and lower craving reactivity after exposure to drug-related cues (Kriegler et al., 2019). Our team also demonstrated that the MMT group showed higher regional homogeneity in the local activity of executive control (i.e. in the dlPFC) in HUDs and that MMT might improve executive control (Xue et al., 2022). High methadone dosages induce heroin tolerance. A review of randomized controlled trials demonstrated that a high dosage was more effective in reducing withdrawal symptoms or negative reactions and prolonging abstinence from heroin (Faggiano et al., 2003). Methadone suppresses the physiological and psychological craving response to heroin in a time- and dosage-dependent mode through the progressive development of tolerance (Bell & Strang, 2020). We speculated that the methadone dosage impacts the bilateral RAI scores and SN-ECN connectivity, leading to a reduced craving for heroin. Additionally, the right RAI scores and SN-rECN might be contributing to a decrease in craving for heroin during the PA program. Based on our findings, the connectivity of SN-rECN and rRAI was negatively correlated with craving intensity. It suggested that the SN-rECN connectivity and rRAI may serve as potential biomarkers for craving, which could be utilized for objectively assessing the severity of addiction and the efficacy of treatments (such as repetitive transcranial magnetic stimulation).

## Limitations

Although our data present robust findings, some limitations should be considered. First, due to recruitment difficulties, only a few females were included in this study. Whether these results could be generalized to females need further studying. Second, we asked all the participants not to think of anything special during the rs-fMRI. However, we cannot be sure they did so. This is a common problem in rs-fMRI. Third, the individual characteristics of brain function remain unclear. Our future research will focus on understanding cognitive performance through individual brain connectivity to provide new insights into the mechanisms of drug addiction. Fourth, during participant recruitment, an experienced psychiatrist conducted face-to-face interviews and urine tests for drug components, ensuring thorough initial screening. However, the absence of some scales like Mini-International Neuropsychiatric Interview may have led to undetected psychiatric comorbidities, potentially affecting the study’s precision and generalizability.

## Conclusion

This study demonstrated that the MMT and PA programs altered SN-rECN coupling in individuals with HUD. SN-rECN connectivity was negatively correlated with craving for heroin in both programs. Importantly, the MMT program enhanced SN-lECN coupling and increased the left RAI scores more than the PA program. Our findings also suggested that the RAI score could predict heroin craving in individuals with HUD undergoing the MMT or PA program.
